# The Conundrum of Adjuvant HER2 Treatment Options

**DOI:** 10.3389/fonc.2018.00177

**Published:** 2018-05-30

**Authors:** Ajaz Bulbul, Emilio Araujo-Mino, Zoneddy Ruiz Dayao

**Affiliations:** ^1^Division of Internal Medicine, Department of Hematology/Oncology, Texas Tech University Health Sciences Center School of Medicine, Lubbock, TX, United States; ^2^Hematology and Oncology, Kymera Cancer Center, Carlsbad, NM, United States; ^3^Division of Hematology Oncology, University of New Mexico Comprehensive Cancer Center, Albuquerque, NM, United States

**Keywords:** HER2, neratinib, APHINITY, trastuzumab, pertuzumab, ExteNet

## Introduction

Improving outcomes in HER2 over-expressing breast cancer has been an impressive success story over the years. Remarkable clinical benefit and large hazard ratios in earlier metastatic and adjuvant trials rendered hope to more contemporary adjuvant trials of a similar large-scale benefit. Recent FDA approvals of dual HER2-blockade with trastuzumab plus pertuzumab and neratinib based on the APHINITY and ExteNET trials, respectively, were modest and rather underwhelming. Future trials need to focus on identifying robust biomarkers and clinical parameters that can best define the subset of patients where the anticipated toxicities and cost of therapy are justified.

## Role of Dual HER2 Blockade in the Adjuvant Treatment

**Figure 1:**
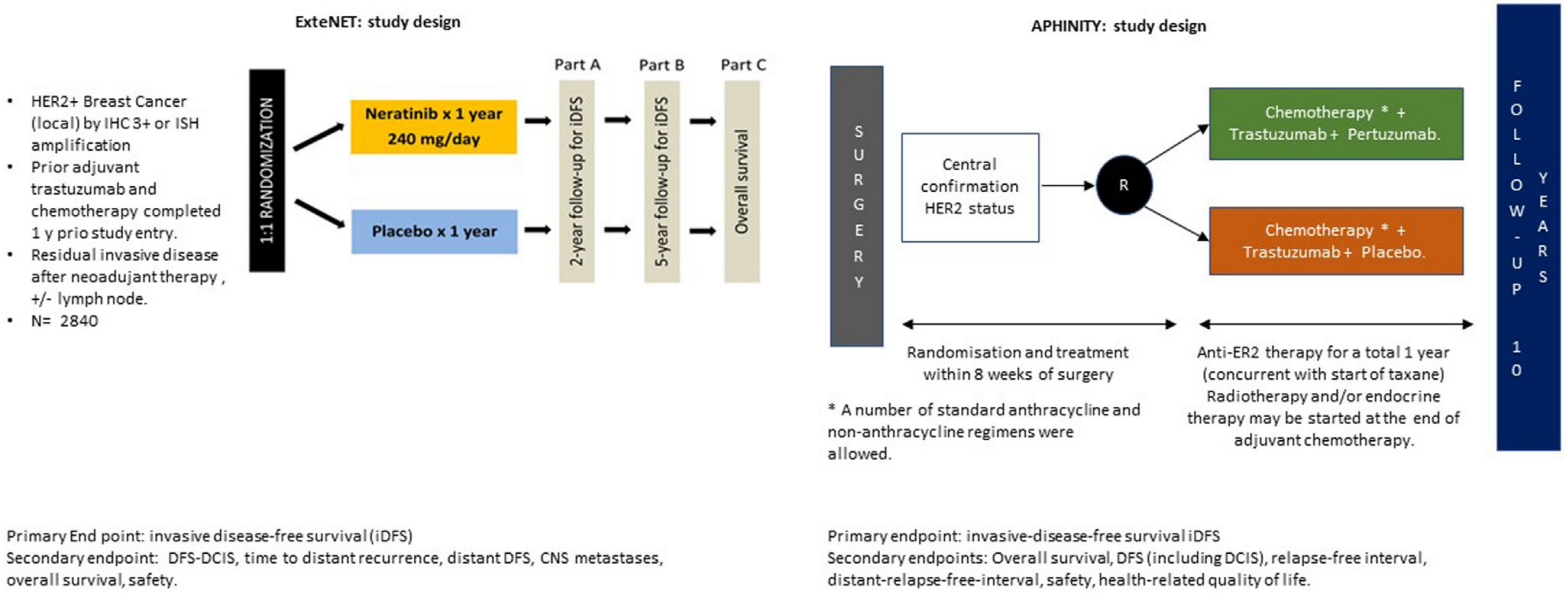
ExteNet and APHINITY trial design.

APHINITY was an adjuvant study of 4,805 HER2-positive post mastectomy or lumpectomy patients randomly assigned to receive standard 18-week chemotherapy plus 1 year of either trastuzumab and placebo or trastuzumab and pertuzumab for tumors > 1 cm (Figure 1).

von Minckwitz et al. (1) and the APHINITY team showed a positive but disappointing 0.9% absolute benefit in invasive disease-free survival (iDFS) (HR 0.81). Even among higher risk node-positive women, pertuzumab improved the 3-year disease-free survival (DFS) by only 1.8%, from 90.2 to 92.0% (HR 0.77, 95%; *P* = 0.02). The modest benefit was mostly confined to the two-thirds of patients who were node positive and roughly the third who had HR-negative disease with an absolute benefit of 1.8 and 1.6%, respectively, with the addition of pertuzumab at 3 years.

The recurrence rate was low in node-negative patients. Not surprisingly, there was no difference between the treatment arms. Similarly, in the HR-positive group, a benefit was not evident. Node-negative enrollment accounted for 36% of patients which is higher than the 12% in the N9831 and B-31 (2), possibly diluting a potential benefit and accounting for the better than expected 93.2% 3-year DFS in the placebo group as compared to 86.7% in the N9831 and B-31 joint analysis (2) at 3.5 years, 88% with BCIRG006 at 3 years (3), and 85.8% at 2 years in HERA (4) and 91.6% a more contemporary ExteNET (5). In addition, APHINITY may have included lower risk patients in the beginning since later the protocol was amended to limit enrollment of high-risk node-negative and allow more node-positive disease patients when 3,655 out of 4,804 were already enrolled (Table 1).

**Table 1:** Selected adjuvant HER2 trials.

Patient characteristics	BCIRG 006	HERA	Joint analysis NSABP B-31 and NCCTG N9831	APHINITY	ExteNet
ER+ (%)	54	50	53	64	57
ER− (%)	46	50	47	36	43
T1 (%)	40	39	41	40	31
T2 (%)	54	44	43	53	40
T3 (%)	6	5	14.4^a^	6	9
N0 (%)	28	32	13	38	24
N1 (%)	38	29	48	37	47
N2 (%)	23	28	24	25	30
N3 (%)	10	NR	13	NR	NR
**Chemo regimen**					
Control arm	AC-T	Several chemo neo/adj	AC-T	Chemo + T	Several chemo + T neo/adj
Experimental arm	AC-T + 1 year trastuzumab (Tz)^b^	Chemo + 1 year Tz^c^	AC-T + 1 year Tz	Chemo + Tz + P	Chemo + Tz + neratinib
Disease-free survival (DFS) (%)	3 years: 81 vs 88	2 years: 87.4 vs 85.8	3 years: 75.4 vs 87.1	3 years: 93.2 vs 94.1	2 years: 91.6 vs 93.9
	5 years: 75 vs 84	4 years: 73 vs 78.3	4 years: 67.1 vs 85.3		
Hazard ratio for DFS	5 years: 0.64	4 years: 0.76	3 years: 0.48	3 years: 0.81	2 years: 0.67
Overall survival (%)	5 years: 87 vs 92	4 years: 87.4 vs 89.3	3 years: 91.7 vs 94.3	NR	NR
Hazar ratio for OS	5 years: 0.63	4 years: 0.85	3 years: 0.67	NR	NR

*Staging, nodal status, and respective DFS*.

*^a^Tumors > 4 cm*.

*^b^TCH evaluated but not included in this table*.

*^c^2-Year trastuzumab was also evaluated*.

*NR, not reported*.

We need to recognize that patients in this trial overall had an excellent prognosis as is expected now with contemporary adjuvant trials with standard chemotherapy/trastuzumab treatment. Large benefits are difficult to achieve, and many need to be treated to benefit a few. As an example, the APT trial in node-negative patients had an excellent 3-year DFS of 98.7% with only weekly paclitaxel with a year of trastuzumab dual blockade. It would be difficult to improve on these numbers in this cohort of low-risk patients (6).

Eight-year data from HERA trial showed the HR for DFS of 0.76 which was similar at 4 years and attenuated compared to the HR 0.54 at year 1 suggesting that there is a possibly diminishing benefit with a flattening of the DFS curve after an initial drop (4). These results are consistent with the magnitude of benefit seen when lapatinib was added to trastuzumab in the ALLTO trial (86 vs 88% 4 years DFS) (7). In our opinion, therefore, it is unlikely that longer follow-up would magnify the benefit currently reported; except for possibly the high-risk node-positive patients; which would merit future follow-up of the trial.

The challenge lies in identifying who can benefit from therapy and avoid overtreatment. Future studies need to focus on evaluating molecular markers that elucidate the heterogeneous response to anti-HER2 agents and predict responses. Unfortunately, the biomarker analysis in TRYPHAENA (8), CLEOPATRA (9), and NeoSphere (10) were mostly negative with PIK3CA being mainly prognostic but not predictive and with limited power to detect correlations. High HER2 protein, HER2, and HER3 mRNA levels, wild-type PIK3CA, and low serum HER2 showed a significantly better prognosis (*P* < 0.05) (9). However, other trials show promising molecular signals. In the docetaxel, trastuzumab, pertuzumab arm of the NeoSphere trial, higher expression of immunogenic markers PD1 and STAT1, CTLA4, MHC1 were linked with lower pathological CR rate suggesting that the combination of anti-HER2 and immune-checkpoint inhibitors (11) is a reasonable strategy to explore. The magnitude of CD8+ tumor-infiltrating lymphocytes in TNBC and HER2 overexpressing tumors may help identify breast cancers with higher predicted response rates (12). Novel prognostic markers are similarly being explored. Higher anti-HER2 CD4+ T-helper type 1 response is a promising immune correlate to pathologic response. HER2-directed Th1 immune interventions like HER2 dendritic cell vaccine have shown mitigation of pCR rates in early studies (13). The PANACEA trial (NCT02129556) is currently evaluating this concept in the metastatic setting with trastuzumab and NCT03032107 with TDM-1. A CLEOPATRA’esque NCT03199885 design is evaluating pembrolizumab with paclitaxel and dual HER2 blockade.

Traditionally, identification of HER positivity is made by identification of overexpression or amplification of the HER2 gene (14). In the NSABP-47 trial, there was no benefit noted with trastuzumab in the low HER2 expressing tumors (IHC 1,2+, FISH < 2). These validate that trastuzumab has no current role in the HER2-negative population as currently defined. However, somatic mutations in the HER2 gene, HER3, HER4 which may not be identified by traditional testing are sometimes functionally active mutations. It is unclear if these can be potentially targeted by current treatments (15, 16).

Adaptive immune mechanisms may have a role in modulating pCR with HER2-directed therapy in high PDL1 expressers (17). RNA sequencing data from NeoALLTO suggest that pCR was associated with high expression of *ERBB2* and low expression of *ESR1* (estrogen receptor 1) across arms and more importantly high expression of immune gene signatures and low expression of stroma gene signatures were predictive of higher PCR (18). With more sequencing data available, correlative studies should pave for enriching future treatment studies for these patient populations. This would be invaluable for optimization of patient treatment.

## Role of Neratinib in Adjuvant Treatment

ExteNET enrolled 2,840 HER2-positive patients who had received standard chemotherapy followed by 1 year of maintenance trastuzumab. These patients were considered high-risk owing to their node positivity or residual disease post neoadjuvant therapy. Patients in the treatment arm received 1 year of neratinib 240 mg/day (Figure 1).

Results of the ExteNET trial suggest an overall benefit of neratinib with a 2.3% absolute improvement in the 2-year iDFS (5). The trial underwent amendment to limit enrollment to node-positive patients (after it already included 24% node negative in the analysis of the study group). Concordant with the HERA trial where there was an early separation on the iDFS curves in the second year of extended trastuzumab therapy in both hormone positive and negative patients; a similar 2-year separation of curves was observed in the ExteNET trial. However, the benefit was primarily seen in the hormone positive patients (95.4 vs 91.2%, *P* = 0.0013). Neratinib showed a 2.3% absolute difference in DFS compared to placebo after 2 years, with stratification for hormone receptor status showing a pronounced HR of 0.51 for ER-positive disease, perhaps resulting from modifying estrogen receptor sensitivity to hormonal agents. Patients with ≥4 positive lymph nodes also achieved an additional benefit reflected in a superior DFS of 97.8 vs 96.5% at 2 years with HR of 0.65 (0.41–1.01) (5).

These findings support a potential benefit of “pan HER inhibition” in the HR-positive population. Barring the high rate of diarrhea, this TKI would seem a likely next candidate in the HER2 saga, primarily in HR-positive disease. Witton et al. have shown there is a strong interaction, in terms of survival, between HER expression and ER expression and in the HER2-positive tumors, with the curve flattening after 6 years (19). However, whether this translates to a survival improvement is not clear. Neoadjuvant chemotherapy was received in 24% in the neratinib group and 27% in the placebo, and no separate analysis was done to only assess patient with neoadjuvant treatment, but they were included in the overall analysis and benefit.

MAPK pathways may be downregulated by neratinib, and the activity against AKT and ERK could be correlated with efficacy in neratinib (20, 21), but these need to be studied in robust correlative studies. Biomarker candidates who have been investigated *in vitro* for neratinib include upregulation of RB1CC1, HER3, FOXO3a, and NR3C1, as well as downregulation of CCND1 mRNA (14, 22).

Taken in context, however, one should not forget the cost issue. The addition of pertuzumab will add approximately US$70,000/year. The estimated yearly cost of neratinib is US$120,000. In addition to the cost of treatment, the clinical toxicity including cardiac toxicity and diarrhea must be considered. Grade 3 diarrhea was seen in up to 40% patients. The median duration of diarrhea was 5 days and occurred very early mostly during the first month. A protocol amendment mandating antidiarrheal prophylaxis with loperamide in tapering down fashion reduced the grade 3 diarrhea to 17% in exploratory analysis (5).

For any future studies involving a population with good prognosis, the challenge is to identify the few that benefit from therapy and the majority that do not. As a matter of perspective, other trials in the non-HER2 expressing population have paved the way for fine tuning treatment. Studies on gene expression profiling have established risk categories that identify patients who will have no benefit from chemotherapy. The development and validation of prognostic and predictive tools for the selective use of dual therapy in HER2-positive breast cancer would be a big step in the care of these patients. We need to identify candidates for either drug in practice setting since currently, no comparative data are available to facilitate this decision (Table 2).

**Table 2:** Patients benefiting in APHINITY and ExteNET.

Patient characteristic	Neratinib	Pertuzumab
DFS	95.2 (HR+) vs 91.2% (HR−) DFS@ 2 years	92 vs 90% 3 years iDFS
Node positive	HR 0.70 in node + similar to ITT population^a^	1.8% Absolute DFS improvement
HR+	HR 0.51 for HR+ (improved outcome)	HR 0.81 (overall); 0.77 in high-risk node positive
HR−	No benefit	1.6% Absolute DFS improvement
Mechanism	PAN HER inhibition, MAPK, ERK, AKT downregulation	HER2 inhibition
Biomarker candidates	RB1CC1, HER3, FOXO3a, NR3C1, CCND1	CD8 TIL, anti-HER2 CD4+ T helper, high HER2 protein, HER2 and HER3 mRNA levels, PD1 for addition of IO
Ideal patient	High risk, node positive, HR+	High risk, node positive, HR−
Absolute DFS improvement	2.3% Absolute DFS improvement @ 2 years	0.9% Absolute iDFS improvement @ 3 years^a^
Cost (USD)	$120,000/year	$70,000/year

*Ideal patient candidates for adjuvant neratinib vs pertuzumab in clinical setting outside of a clinical trial given no comparative studies between the drugs. These are considerations in HER2-positive high-risk patients*.

*HR, hormone receptor; DFS, disease-free survival; ITT, intention to treat; TIL, tumor-infiltrating lymphocytes; iDFS, invasive disease-free survival*.

*^a^2-Year. DFS based on LN status LN neg: 99.4 vs 99.2%, HR 0.82 (0.32–2.03); LN 1–3: 97.8 vs 96.5%, HR 0.66 (0.41–1.02); LN ≥ 4: DFS 97.8 vs 96.5%, HR 0.65 (0.41–1.01)*.

## Conclusion

The development of HER2 targeted agents resulted in dramatic survival benefits when added to a chemotherapy backbone, a significant stride in the treatment of what was once considered a poor prognosis subgroup. However, it is expected that any additional benefit from newer HER2-targeted agents will provide smaller incremental benefits.

FDA approval of these new HER2-targeted agents does not mean that every patient is a candidate for these treatments. While we find comfort in the growing number of therapeutic options that we once did not have, our enthusiasm in prescribing these newer class of drugs must be balanced with financial pragmatism, anticipated treatment-related toxicities, and a realistic estimation of the degree of benefit for the individual patient. In this regard, the optimal patient selection is still lacking.

Future adjuvant HER2 trials need to use selected rather than unselected populations and should be focused on using less intensive therapy when possible, and on finding biomarkers on secondary analysis. We need to be careful in the presumption that benefits in metastatic disease setting can be translated to the adjuvant setting especially in an unselected population (23, 24). For now, we should probably be restricting the use of adjuvant pertuzumab to women with node-positive disease, and considering neratinib in the high risk, hormone receptor-positive population.

## Author Contributions

AB—concept and manuscript writing. EA-M and ZD—manuscript writing.

## Conflict of Interest Statement

AB is on advisory board of Pfizer and Astra Zeneca. The remaining authors declare that the research was conducted in the absence of any commercial or financial relationships that could be construed as a potential conflict of interest.
